# Trends in Indications and Techniques of Corneal Transplantation from 1999 through 2015 at a Tertiary Referral Center in Athens, Greece

**DOI:** 10.1155/2018/9132083

**Published:** 2018-07-18

**Authors:** Konstantinos Droutsas, Georgios Bagikos, Dimitrios Miltsakakis, Ilias Georgalas, Apostolos Lazaridis, Klio Chatzistefanou, Marilita M. Moschos, Chryssanthi Koutsandrea, Georgios Kymionis

**Affiliations:** ^1^First Department of Ophthalmology, National and Kapodistrian University of Athens, Athens, Greece; ^2^Department of Ophthalmology, Philipps University, Marburg, Germany; ^3^State Ophthalmology Clinic, General Hospital “G. Gennimatas”, Athens, Greece; ^4^Jules Gonin Eye Hospital, University of Lausanne, Lausanne, Switzerland

## Abstract

**Introduction:**

During the past decade, novel techniques of corneal transplantation allowing faster and better restoration of vision have emerged. The present cohort study describes a shift of indications and techniques that has occurred in the field of corneal transplantation over a 17-year period in Greece.

**Methods:**

All patients undergoing keratoplasty between January 1999 and December 2015 at an academic tertiary referral center in Athens, Greece, were retrospectively reviewed. The annual incidence of keratoplasty indications and techniques was recorded and analyzed.

**Results:**

A total of 1382 keratoplasty procedures were included. Leading indications were bullous keratopathy (BK) (37.5%), followed by allograft rejection (17.7%), corneal scar (12%), keratoconus (KC) (10.3%), and Fuchs endothelial dystrophy (FED) (8.8%). A decreasing trend was observed for KC (*P*=0.009) and an increasing trend for BK (*P*=0.003) and FED (*P*=0.001). In 2015, the incidence of penetrating keratoplasty (PK) had decreased from 100% (1999 to 2009) to 21.4%; for cases with isolated pathology of the corneal endothelium, DSAEK was the preferred technique (59.8%), while the respective rate of DMEK was 18.8%.

**Conclusion:**

Herein, we observed an increasing trend of endothelial pathology among keratoplasty indications as well as a major shift in preferred techniques due to a wide adoption of the new EK procedures.

## 1. Introduction

Keratoplasty is one of the most common and successful allotransplantations in humans and the only treatment of several corneal pathologies. Since the first successful corneal transplantation by Zirm in 1906 [1], penetrating keratoplasty (PK) was the gold standard. Over the past decade, however, advancements in the field of corneal transplantation changed the preferred practice patterns of corneal surgeons. The more or less selective transplantation of corneal endothelium evolved since the introduction of posterior lamellar keratoplasty in 1998 [2] to safer and more targeted techniques, for example, Descemet (automated) stripping endothelial keratoplasty (DS(A)EK) [3] and Descemet membrane endothelial keratoplasty (DMEK) [4, 5]. Moreover, advancements in the field of keratoconus surgery that may delay or even arrest disease progression, that is, corneal crosslinking (CXL), have reduced the need for keratoplasty [6].

Indeed, recent reports describe both an increase of endothelial pathology and a shift from PK to lamellar keratoplasty techniques [7–9]. However, only limited data on the implementation of DMEK among keratoplasty procedures are available [8, 9]. Purpose of the current paper is therefore to report trends in indications and techniques in a cohort where both DMEK and DSAEK were implemented.

## 2. Materials and Methods

The records of all corneal transplantations conducted between 1999 through 2015 at a referral center in a large hospital in Athens, Greece (First Department of Ophthalmology, National and Kapodistrian University, Athens, and Ophthalmology Department, General Hospital “G. Gennimatas”) were reviewed.

All procedures were performed by 3 surgeons in total. The number of surgeons operating during each calendar year varied from 1 to 3.

The present research adhered to the tenets of the Declaration of Helsinki. Due to the observational and retrospective character of the study, IRB approval was not required.

### 2.1. Data Collection

Indications were grouped according to the following criteria:

1. “Bullous keratopathy” (BK) included all cases of corneal endothelial decompensation except for cases of Fuchs endothelial dystrophy and failed corneal grafts.
2. “Regraft” included cases of repeat keratoplasty due to graft failure.
3. “Corneal scar” included stromal opacities, for example, after herpetic and infectious keratitis, pemphigoid, chemical and mechanical trauma, trachoma, or anesthetic abuse.
4. “Keratoconus” (KC).
5. “Fuchs endothelial dystrophy” (FED).
6. “Sterile melt/perforation” included all cases of noninfectious keratitis leading to corneal perforation.
7. “Infectious keratitis” included all cases of therapeutic keratoplasty for progressive corneal melt due to bacteria, fungi, or acanthameba. This group also included cases where the pathogen could not be isolated.
8. “Stromal dystrophies” (SD).

### 2.2. Statistical Analysis

All data were collected with Excel software (version 14, Microsoft Corp.). Descriptive and inferential data analysis was performed with SPSS software (version 17.0, SPSS Inc.). Numerical data are presented as mean ± standard deviation. Evolution of recipient age, gender, and surgical indication were assessed using linear regression analysis. The *x*^2^ test was used where appropriate. A *P* value less than 0.05 was considered statistically significant.

## 3. Results

### 3.1. Demographics

The present retrospective cohort study includes 1382 eyes undergoing corneal transplantation at a single tertiary referral center with a nationwide patient pool. While the majority of donor tissue (79.4%) was imported from eye banks outside Greece, the rest was retrieved from local multiorgan donors.

**Figure 1:**
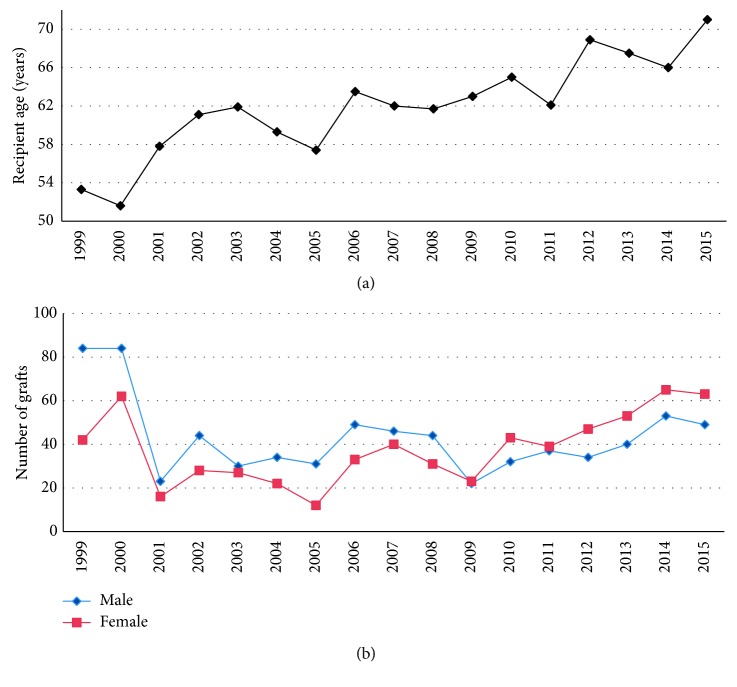
Chart depicting distribution of recipient age (a) and gender distribution (b) per calendar year. (a) Recipient age shows a statistically significant increase (*P* < 0.001, *r*^2^=0.79, univariate linear regression). (b) In addition, a switch from male to female predominance is noted in 2009, the year of EK implementation. This also may be explained by the increase Fuchs endothelial dystrophy that affects mainly women.

Mean recipient age was 61.9 ± 5.1 years with increasing trend, starting at 53.3 ± 20.7 years (1999) and reaching 71.1 ± 12.0 years (2015) (*P* < 0.001, *r*^2^=0.79, univariate linear regression) (Figure 1(a)).

The present cohort comprises 736 men (53.3%) and 646 women (46.7%) in total. In 2009, a switch of male to female predominance was evident, suggesting an increasing trend for female patients. Indeed, when dividing the observation time into a first (1999 through 2008) and last period (2009 through 2015), an increasing trend for females was found only in the last period (*P*=0.011, *r*^2^=0.89, univariate linear regression) (Figure 1(b)).

**Figure 2:**
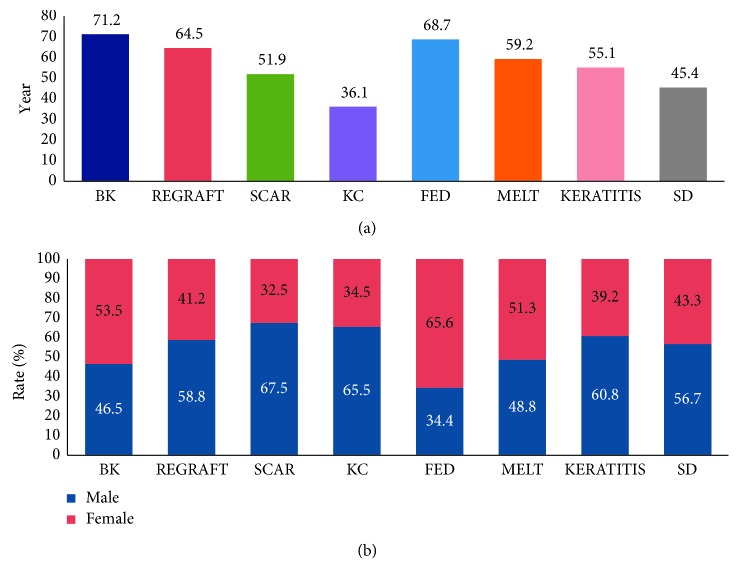
Chart depicting recipient age (a) and gender distribution (b) per indication. (a) The lowest age is observed in keratoconus while the highest in Fuchs endothelial dystrophy patients. (b) Significant predominance of men is noted in keratoconus (65.5%) and corneal scar (67.5%) whereas of women in Fuchs endothelial dystrophy patients (65.6%).

Patients with keratoconus (KC, mean age 36.1 ± 14.3 years) formed the youngest and patients with BK the oldest group (mean age 71.2 ± 11.5 years) (Figure 2(a)). A significant predominance of men was found in KC (*x*^2^=13.36, *P* < 0.001), regraft (*x*^2^=7.55, *P*=0.006), and scar (*x*^2^=20.27, *P* < 0.001), while that of women was found in FED (*x*^2^=11.84, *P*=0.001) (Figure 2(b)).

### 3.2. Indications

**Figure 3:**
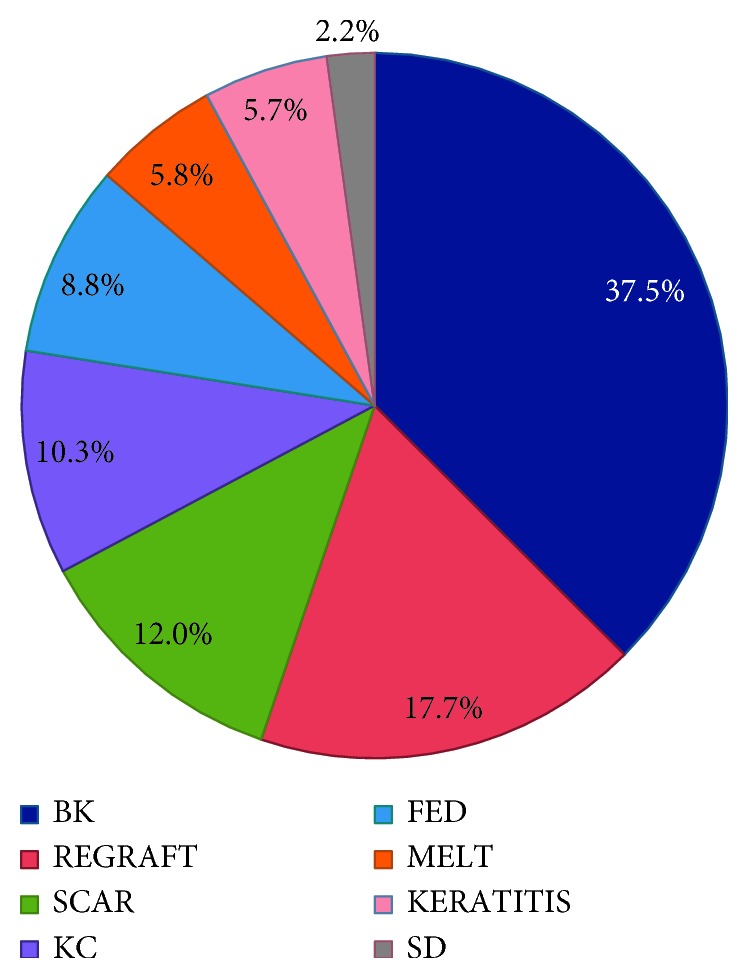
The pie chart illustrates the incidence of all keratoplasty indications from 1999 to 2015. Top 3 indications were bullous keratopathy (37.5%), regraft (17.7%), and corneal scar (12%). Each diagnosis is represented by the same color as in the bar chart in Figure 2.

Leading indication was BK (37.5%), followed by allograft rejection (17.7%), corneal scar (12%), KC (10.3%), FED (8.8%), corneal melt (5.8%), infectious keratitis (5.7%), and stromal dystrophy (2.2%) (Figure 3).

**Figure 4:**
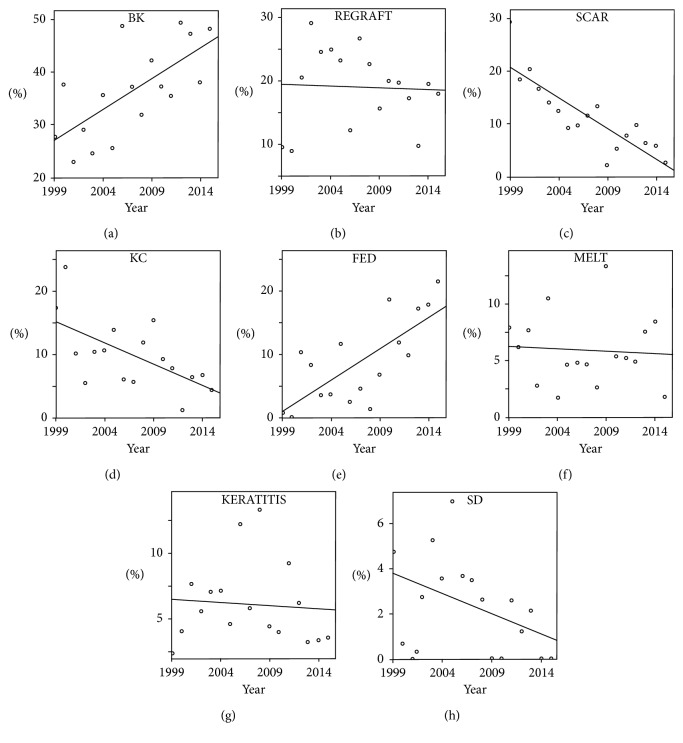
Scatter plot depicting the annual incidence of each keratoplasty indication. A significant trend was found for bullous keratopathy (BK; *r*^2^=0.47, *P*=0.003), corneal scar (*r*^2^=0.72, *P* < 0.001), keratoconus (KC; *r*^2^=0.38, *P*=0.009), and Fuchs endothelial dystrophy (FED; *r*^2^=0.55, *P*=0.001). All others were found not significant (univariate linear regression analysis).

Univariate linear regression analysis of the annual incidence for each indication revealed a significant increase of endothelial pathologies, that is, BK (*r*^2^=0.47, *P*=0.003) and FED (*r*^2^=0.55, *P*=0.001) and a significant decrease in corneal scar (*r*^2^=0.72, *P* < 0.001) and KC (*r*^2^=0.38, *P*=0.009). All other indications did not show significant fluctuations (univariate linear regression analysis) (Figure 4).

### 3.3. Techniques

**Figure 5:**
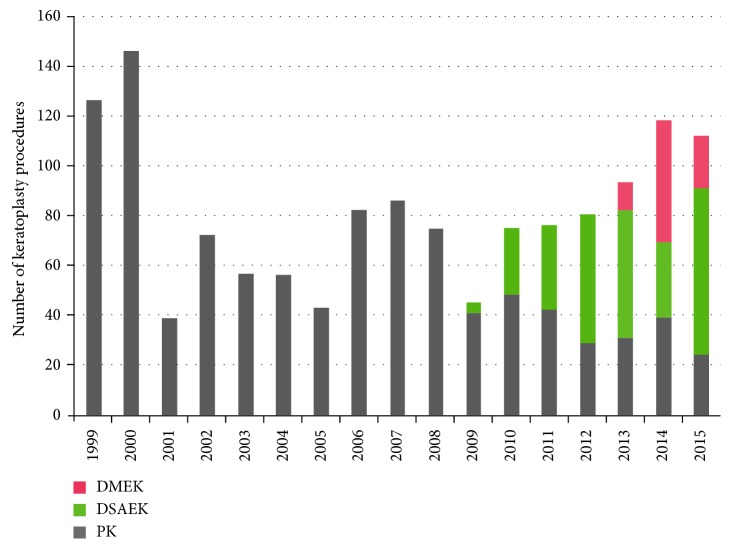
Column chart depicting the absolute number of keratoplasty procedures performed in each calendar year from 1999 to 2015. Penetrating keratoplasty (gray columns) was the only technique applied until DSAEK (green columns) and DMEK (red columns) were implemented (2009 and 2013, resp.). In the last year, PK was only performed in 21.4%. Significant fluctuations in the annual number of surgeries were caused by fluctuations in the number of operating surgeons as well as by graft shortage periods.

PK was the only technique applied until 2009, when DSAEK was introduced, starting with 8.9% (2009) and reaching 59.9% (2015) (Figure 5). DMEK was introduced in July 2013 reaching 11.8% in the first (half) year, peaking with 41.5% in the second year, and decreasing to 18.8% in the third year of its implementation (Figure 5).

**Figure 6:**
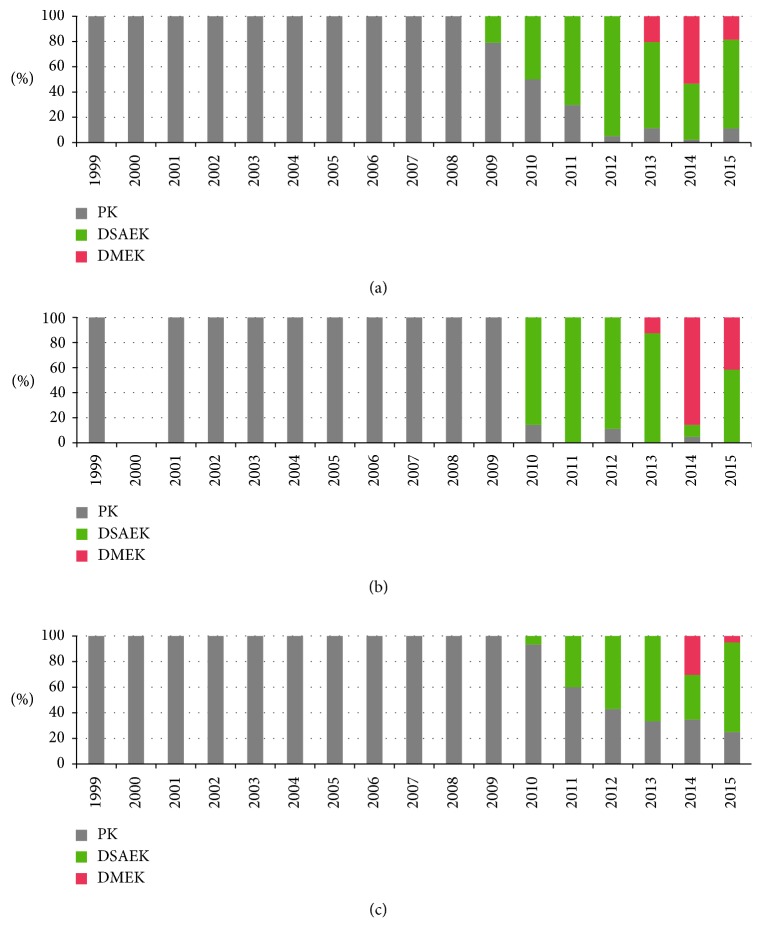
Column chart depicting keratoplasty techniques applied for endothelial pathology, that is, BK (a), FED (b), and regrafts (c). Note that, in the last 3–6 years of the observation period, PK has been replaced to a variable extent by endothelial keratoplasty, that is, DSAEK and DMEK.

By the final year of the study, EK was the preferred treatment for BK (89.8% EK versus 11.2% PK) and the only treatment for FED (Figure 6).

## 4. Discussion

The present study reports trends in keratoplasty indications and techniques from 1999 through 2015 at an academic tertiary referral center in Athens, Greece. As part of this study, the implementation of new endothelial keratoplasty techniques (DMEK and DSAEK) in a large keratoplasty cohort was evaluated.

The leading indication herein was BK (37.7%), being in accordance with a previous Greek multicenter study reporting aphakic/pseudophakic corneal edema (29.1%), keratoconus (26%), and regraft (11.9%) as main indications [10].

The increase of corneal endothelial pathology (BK and FED) among indications may be explained by the early and successful adoption of DSAEK and DMEK in our cohort (Figure 4).

On the other hand, KC demonstrated a significant decrease over time that may relate to the introduction of CXL as well as advancements in optical rehabilitation, for example, scleral contact lenses or intracorneal ring segments.

Compared to the global introduction of new lamellar techniques [7], the present cohort keeps up with advancements in endothelial keratoplasty. Thus, although it took three years for EK (DSAEK) to surpass PK, both EK techniques (DSAEK and DMEK) were successfully implemented, with EK accounting for 78.6% of the total keratoplasty procedures performed during the final year of our study (Figure 5). This is in agreement with other studies reporting similar trends in keratoplasty techniques [7–9]. With regard to endothelial pathology, EK procedures have almost completely replaced PK, ranging between 88.9% for BK and 100% for FED cases (Figure 6).

This rapid shift in surgeons' preferences from PK to EK techniques in the treatment of endothelial pathology can easily be explained by several advantages of EK, for example, smaller incisions, fewer or no sutures, and no open sky surgery. The main advantage of EK however is faster and better visual rehabilitation and lower risk for immunologic rejection [8, 11, 12].

Another notable observation is the smooth implementation of DMEK accounting for 36% of EK procedures during the last 3 years of the study, as opposed to a peak rate of 11% for 2014, as recently reported by the Eye Bank Association of America [7].

In conclusion, keratoplasty indications showed remarkable trends over the past 17 years. These changes relate to the introduction of endothelial keratoplasty as well as to new treatment alternatives for KC other than keratoplasty. Respectively, a considerable shift of keratoplasty techniques from PK to DSAEK and DMEK in the treatment of endothelial disorders is evident. Finally, despite being a challenging procedure, DMEK was successfully implemented in our center.
